# Market failure, policy failure and other distortions in chronic disease markets

**DOI:** 10.1186/1472-6963-9-102

**Published:** 2009-06-18

**Authors:** Jennifer J Watts, Leonie Segal

**Affiliations:** 1 Centre for Health Economics, Monash University, Building 75, Melbourne, Australia; 2 Health Economics & Policy Group, Divn Health Sciences, GPO Box 2471 University of South Australia, Adelaide, Australia

## Abstract

**Background:**

The increasing prevalence of chronic disease represents a significant burden on most health systems. This paper explores the market failures and policy failures that exist in the management of chronic diseases.

**Discussion:**

There are many sources of market failure in health care that undermine the efficiency of chronic disease management. These include incomplete information as well as information asymmetry between providers and consumers, the effect of externalities on consumer behaviour, and the divergence between social and private time preference rates. This has seen government and policy interventions to address both market failures and distributional issues resulting from the inability of private markets to reach an efficient and equitable distribution of resources. However, these have introduced a series of policy failures such as distorted re-imbursement arrangements across modalities and delivery settings.

**Summary:**

The paper concludes that market failure resulting from a preference of individuals for 'immediate gratification' in the form of health care and disease management, rather than preventative services, where the benefits are delayed, has a major impact on achieving an efficient allocation of resources in markets for the management of chronic diseases. This distortion is compounded by government health policy that tends to favour medical and pharmaceutical interventions further contributing to distortions in the allocation of resources and inefficiencies in the management of chronic disease.

## Background

Chronic disease is a major cause of morbidity in most high income countries, consuming approximately 70% of health care expenditure in the US [1]. The World Health Organisation (WHO) in 2002 estimated that chronic disease would account for 72% of the global disease burden, with 35 million deaths or approximately 60% of total deaths worldwide attributed to chronic disease [2]. The increase in the prevalence of chronic disease has been a pattern noted in most developed countries since the 1970s, and this trend is likely to continue with population ageing. Despite evidence of increasing prevalence of chronic diseases and ageing populations, health systems have supported reactive management to acute illness, injury and acute exacerbations of established diseases, rather than primary and secondary disease prevention [3-5]. Funding arrangements, including health insurance tend to be focused on hospital and medical care and pharmaceutical management distorting care towards these modalities [6].

Whilst health care markets are routinely described as not conforming to the conditions required for competitive markets, when analysing the competitiveness or features of separate markets within the health care system, there are differences with respect to the degree of divergence from the assumptions required for competitiveness. Such differences are especially apparent when comparing markets for acute health care to those for chronic disease management. Differences particularly relate to the role of information, including the degree of information asymmetry between providers and consumers; and the role of intertemporal factors and the associated rate of time discount that create a wedge between individual and social preferences for health care.

Market failures, inefficiencies and distributional issues (equity) are the primary reasons for government intervention in the health care market. Governments intervene through direct provision of services, including direct funding to public or private bodies for the provision of health services; subsidy to consumers for private health services; or subsidies to consumers for the purchase of private health insurance. In general government intervention in any market has flow-on effects to other parts of the market or related markets, which can create further distortions. The market distortions that arise from these flow-on effects can be termed 'policy failure'. In the health care sector policy failure is widely observed and arises for instance where funding or subsidy of services occurs irrespective of evidence of cost effectiveness and contribution to other societal objectives.

The objective of this paper is to describe the important market and policy failures present in chronic disease markets, and to provide an overview of policy options to address these sources of failure.

## Discussion

### Information asymmetry or the 'expert patient'?

Information asymmetry between providers and consumers has long been accepted as a feature of health markets, and is widely recognised as a cause of market failure [7-9]. The imperfect agency relationship that arises is a possible contributor to excess demand for health care (also termed 'supplier induced demand'), where the provider has an income or financial incentive to promote excessive health care use [10]. Whilst this is a potential issue for all health markets, the degree to which imperfect agency is likely to distort patterns of consumption is likely to differ across health care markets.

Information in health care necessary for informed or rational decision making by consumers relates to the effectiveness of a particular intervention or service provider. Effectiveness can be judged from a population or societal perspective; or from an individual's perspective. Whilst consumers in all health care markets might not be expected to have information pertaining to clinical or cost effectiveness at the population level, given the choice from a range of interventions, consumers may have adequate information to make an informed choice to meet their own individual needs. In relation to chronic diseases, it is increasingly the case that consumers with established chronic disease may be at least as informed as the clinician in relation to management options relevant to their individual needs, whilst the opposite is almost certainly true in relation to acute care. Given the ongoing nature of chronic disease, it is likely that consumers over time will gain considerable knowledge of their disease, including management options, so they would be well placed to make an informed choice with respect to both their provider and primary care treatment.

Approaches to chronic disease management increasingly refer to the consumer as a 'partner' in their disease management [1,11-13]. Indeed Wilson recognises a consumer's own knowledge base in contributing to successful chronic disease management in the following statement, 'by living with and learning to manage a long term illness many people develop a high degree of expertise and wisdom' [[11];p771]. This recognition of the 'expert patient' in the management of chronic disease has lead to what has been termed a 'partnership model', in which people with a chronic condition play an active role in making decisions about their own health care [11-13]. However funding and delivery arrangements that fail to recognise the consumer's own knowledge with respect to their disease don't support a partnership model and hence are likely to result in treatment and management decisions that are not compatible with maximising individual and population health outcomes [14].

### Time inconsistent preferences and consumption of services in chronic disease management

The argument for discounting future health benefits at the same rate as that of financial resources in private capital markets has support in the economic literature. Olsen suggests however that it is possible that individuals have a different time preference for health compared to ordinary consumption goods and secondly, that an individual's time preference for their own health may differ to that which they attribute to a social intertemporal preference for health [15]. It is also likely that an individual's time preference for their own health will differ according to whether they are valuing an intervention with an immediate gain compared to one that has a future (and less certain) gain. Although consumers with a chronic condition may be able to value services and interventions from which they derive an immediate benefit, their capacity to recognise and value benefits that accrue into the future is less certain. That is an individual consumer is likely to place a lower value, or excessively discount, the downstream benefits that derive from consumption of preventative services compared to the value they place on consumption that derives an immediate benefit. This may arise from a myopic defect as described by Pigou [in [16]], an individual's preference for life-saving interventions as opposed to health improvements [17], or the cost of time to the consumer in primary and secondary prevention.

A discrepancy between an individual's future time preference and the social intertemporal preference rate for health [15] has implications for resource allocation in the management of chronic disease. Where the difference relates to that of 'population uncertainty'; that is the future benefit will accrue to the 'statistical' or unknown persons versus the known individual, current resource allocation decisions may not be consistent with socially efficient allocation over time. That is an individual with a chronic disease may excessively discount future benefits in determining their current consumption decisions, compared to a socially optimal level of consumption. Social benefits from health care, including those externalities that are derived from reducing future mortality and morbidity and any associated reduction in need for high cost care such as hospitalisation, will be maximised with an optimal consumption of primary and secondary preventative care by an individual, particularly in the early stages of chronic disease. It is unlikely however that an individual's current private consumption decisions will reflect the externality or future social health benefit attributable to preventative care.

Social and private benefits from preventative care will almost certainly differ. Benefits from health care, including effective preventative care that reduce mortality and morbidity and associated costs of care (formal and informal) accrue to both the wider society as well as the individual. Consider for instance the externality or impact on others of an individual who has a stroke that may have been avoided. If the impacts on others are not taken into account in the individual's health behaviours, then their current consumption of preventative services will not be at the socially optimal level.

Issues of intergenerational equity may also arise where future generations have to compete for access to health care resources. People with advanced disease may require considerable health care resources that may have been avoidable in part through the optimal consumption of preventative services. Future generations may ultimately carry the burden arising from the under-consumption of preventative services by the current generation.

It can also be argued that individual consumer preferences in relation to the consumption of preventative services might not be rational from an individual's perspective, similar to that of the adoption of harmful behaviours in the rational addiction model. O'Donoghue and Rabin's model of 'time inconsistent' preferences [18] has been used to explain in part the consumption of 'bads', including rising levels of obesity [19]. O'Donoghue and Rabin suggest that a preference for immediate gratification results in the under-consumption of activities with immediate costs and delayed rewards when considered ex-poste compared with ex-ante [18]. This is highly relevant to a consumer's attitude to the prevention and management of a chronic condition and especially the adoption of harmful or unhealthy lifestyle behaviours. The argument of 'immediate gratification' suggests that the market failure is more than just the recognition that consumers prefer gains now to those in the future, solvable with the application of a suitable discount rate. If preferences change over time, the assumption of rational and stable preferences underlying the competitive market model no longer applies and consumer choice, based on ex-ante preferences that differ from ex-poste will not maximise social welfare. Similarly where there is a difference between the individual's and the social marginal rate of time preference, for example due to the existence of a social externality, an individual consumer's choice will result in a loss of social welfare. The divergence between the two may be exacerbated by advertising which, as noted by Moodie and colleagues [19], may actively promote harmful behaviours (such as poor nutrition choices) in relation to chronic disease management and prevention.

In short the assumption of the rational consumer with well-informed stable preferences underlying the competitive market model does not always hold where future benefits are concerned. This is particularly relevant to chronic disease prevention and management, and may be a significant contributor to market failure in chronic disease.

### Market distortion arising from output-based and fee-for-service funding for health care services

Governments have intervened in health care markets in most developed countries in recognition of market failures and equity and distributional considerations. The latter has seen a focus on universal access to basic hospital, medical and emergency services. Whilst this will (and should) continue as a dominant focus of government, changes in population health needs, particularly the increasing prevalence of chronic disease, suggest that government intervention should be concerned with the achievement of allocative efficiency across the health system. However funding models are often focused on achieving technical efficiency, or simple throughput, with no capacity to address the mix of health services. For example, output-based or fee-for-service funding arrangements are oriented towards achieving technical efficiency in a single market, for example medical or hospital markets. The US Medicare system has been described as one geared towards paying for the hospital episode as an acute event, rather than paying for activities that might reduce the need for hospitalisation [6]. In some funding models hospitals are penalised if they invest in activities that reduce the need for hospitalisation even where this represents a cost effective approach to health care.

The nature of the hospital episode has changed, moving towards invasive diagnostic and screening procedures [6] with supply-side moral hazard, arising in the context of fee-for-service payment, and demand side moral hazard, arising out of third party payment (through insurers). Fee-for-service reimbursement also reinforces the silo nature of clinical practice, with clinicians, rather than taking a system-wide view with respect to total resource impact or the patient's overall health, driven by their own activities and incentives they face as individual providers.

As discussed above fee-for-service payment systems provide incentives for health care providers to promote consumer demand for their services, especially where consumers face a zero or subsidised price. The increase in consumption is not likely to be uniform across all services, but will be influenced by the scope of services under the fee-for-service schedule, and also a consumer preference for more immediate benefits and a focus on current health concerns. Preventative primary care services necessary for cost effective management and prevention of chronic disease are likely to miss out [20].

Where payment is based on a single episode of care or service, health care providers have an incentive to increase the number of consultations and reduce their length, as more frequent and shorter consultations tend to support higher incomes. Fee-for-service funding models encourage a focus of care on single health issues, rather than more complex inter-related issues. Depending on the payment schedule, they may encourage additional visits for each health problem, rather than a more holistic approach in which the total health picture is considered. More complex services, such as chronic disease prevention and management, tend to be less conducive to short consultation times and tend to require multi-disciplinary team care, which can be difficult to achieve under fee-for-service, than either a capitated or salary model. Such an incentive pattern is unlikely to promote effective and cost effective chronic disease management. If insurance, or public subsidies favour medical care, as is common, this will compound the distortions.

In relation to tertiary or hospital inpatient care, fee-for-service or output-based payments, where the service is defined as a single hospital separation often based on diagnosis or procedure, will also distort service provision. Where output is defined by diagnosis, providers have an incentive to increase the supply of hospital care. However such an incentive will not apply equally across all hospital services, there is more likely to be an increase in the supply of specific types of hospital care. For instance, hospital care is not homogeneous with respect to degree of risk to the provider. In assessing risk, providers take into consideration the service itself and the characteristics of the patient. In general, procedural care carries a lower financial risk than non-procedural care, as resource inputs are likely to be predictable and thus the provider can make a judgement as to whether the cost is likely to be less than the revenue. Characteristics of patients that are likely to increase financial risk to the hospital include those factors that are likely to influence length of stay, or at least make length of stay difficult to predict. These include age, the existence of co-morbidities and the stage of the patient's disease. The provider's incentive to reduce financial risk is consistent with discrimination against the management of chronic disease in the acute care system.

Technological advances have also been oriented towards diagnosis and procedural capability in the management of acute conditions. For example advances in sameday surgery have increased technical efficiency through new techniques, or newer anaesthetic agents that minimise recovery time and complications associated with surgery. There has been less development in information systems to support integrated and coordinated care management in chronic disease [4]; technological improvement that would be consistent with improving efficiency in the ongoing management of chronic disease.

### Market distortion arising from public subsidies for medical services

Where subsidies are not uniform, but depend on modality or other health service characteristics, the more heavily subsidized service will be promoted. This of course may be the intention, but generally such subsidies are set without regards to the distortions that this will create. Many health systems provide more generous public subsidy for medical and pharmaceutical services than for non-medical services. This is despite evidence suggesting that non-medical services, including allied health services, may be highly cost effective in the prevention and management of chronic disease [21,22]. The price differential created by public subsidy for medical primary care services means that that the price to the consumer without insurance for non-medical services will be high relative to publicly subsidised medical services and pharmaceuticals. This provides an incentive to the consumer to increase their demand for medical services compared to non-medical primary care services. This further enhances the monopoly provision of medical services that is already present by default of practice licensing and registration.

Even if the consumer has private health insurance, non-medical services are likely to be capped unless the insurer has both an incentive and the ability to reduce health care costs, for example, by promoting alternative models of health care delivery (such as case management), to their insured population with chronic disease. Typically however, subsidy regimens mean that allied health services will be underutilised relative to the level of consumption that is optimal for people with chronic disease [6]. Such funding arrangements create distorted incentives potentially redirecting resources away from cost effective care and undermining the role of non-medical primary care services which are critical to the prevention and management of chronic disease.

A further contributing factor to market distortions arising in the primary care setting is the complementarities that arise between medical care and pharmaceutical management. Most ongoing pharmaceutical management requires a prescription from a medical practitioner, thus consumers require regular doctor visits, particularly where drugs have been prescribed for long-term management of chronic disease. Although there is a complimentary relationship that exists between medical and other non-medical services, such as allied health care, they are also substitutable services, and thus potential competitors, particularly in the market for chronic disease management.

### Inequitable distribution of non-medical primary health care services relating to third party compensation

Even within the market for non-medical primary care services there is likely to be an inequitable distribution of services, where entitlement to third party compensation is the driver of demand rather than clinical need. Unlike road traffic and work-place accident victims, people with chronic disease are often not entitled to third party compensation for allied health and other non-medical primary care services, the exception being veterans and holders of private health insurance. Increased demand for these compensated services has resulted in a shift in supply to meet this demand, thus creating a relative shortage of non-medical services. The shortage that has resulted has enabled monopoly pricing, thus the service is targeted to those who are able to pay, or who are eligible for third party funding. Services become even less accessible and affordable to the population with chronic disease or in need of other preventative care. A second effect is that non-medical service providers may focus their effort on services needed by compensated (predominantly accident) victims, thus changing the mix or profile of services that are delivered in the primary care sector away from those needed by people with chronic disease.

### Possible policy responses to market failures in chronic disease management

If the assumption is that consumers with a chronic condition are 'expert patients', information asymmetry is not a significant cause of market failure in the market for chronic disease management. Therefore, supporting consumers to manage their care in partnership with providers, combined with the removal of funding anomalies is necessary to achieve optimal care in chronic disease management and a health service mix across the system more consistent with allocative efficiency objectives.

In the long run it is the price effect resulting from public subsidies for some services and not others that is likely to be the major influence on underutilisation of non-medical primary care and preventative services, rather than a consumer's preference for acute care over health promotion or secondary prevention. If the price for preventative services was subsidised to the same degree as medical services and pharmaceutical products there would be less market distortion created by differential subsidy arrangements. Moreover, ideally the level of subsidy (per service and number of services) should reflect current evidence of cost effectiveness.

Alternative market solutions to chronic disease management could also be considered, particularly those that encourage competition between providers and promote consumer choice. Publicly provided and funded service vouchers, for example through a scheme such as Medicare in Australia, can support consumers to access appropriate care from an allocative efficiency perspective from providers that may not be currently eligible for subsidy [23]. The allocation of service vouchers could be based on evidence of cost effectiveness of particular services or preventative care for chronic conditions and be may be able to provide sufficient incentives for consumers to access appropriate care from their provider of choice. Where patients have knowledge of their chronic disease then enabling them to choose a provider eligible for a subsidy should be consistent with improved health outcomes.

Service vouchers might also help overcome the larger market failure in chronic disease management where individuals are myopic with respect to the consumption of preventative services. Vouchers provide a level of information to the consumer about the services they should be accessing to manage their disease. Consumers would have an entitlement to a given number and type of service vouchers, and, if services are fully subsidised, the price effect is removed from the consumption decision [23]. Other total system options include the adoption of risk-adjusted capitated fund-holding models, which promote flexibility in the service response.

## Summary

Government intervention in health care markets through funding and other mechanisms to address various sources of market failure and inequity in the distribution of health care and health outcomes is widely accepted. However, where governments have intervened by subsidising some types of services and service providers over others, irrespective of evidence of cost effectiveness, this will reduce the ability of the market to achieve efficiency. This is an issue for the management of chronic disease where government subsidy has favoured acute care, including acute exacerbations of chronic disease, and medical services rather than primary and secondary preventative services that are more consistent with the cost effective management of chronic disease [5]. These distortions are exacerbated by fee-for-service payment systems that provide incentives for health care to be technically efficient at the level of a single provider, however provide few incentives for socially efficient delivery.

Resource shifts from acute care markets to markets for the primary and secondary prevention of chronic disease, supported through alternative and flexible funding arrangements, are likely to be consistent with achieving allocative efficiency across the health system. With the increasing prevalence and burden associated with chronic disease it is important that these issues are addressed. Alternative funding mechanisms such as service vouchers and fundholder or capitation models may overcome some of the anomalies that have developed over time between the provision of acute services and chronic disease management, supporting health system objectives of equity and efficient allocation of health care resources.

## Competing interests

The authors declare that they have no competing interests.

## Authors' contributions

JW and LS contributed equally to the ideas in this paper. JW was responsible for drafting the manuscript and preparation for submission to *BMC Health Services Research*. Both authors have read and approved the manuscript.

## Pre-publication history

The pre-publication history for this paper can be accessed here:


